# Supplementary material for: DORA-compliant measures of research quality and impact to assess the performance of researchers in biomedical institutions: Review of published research, international best practice and Delphi survey
Source: PLoS One. 2023 May 12;18(5):e0270616. doi: 10.1371/journal.pone.0270616 (PMC10180594; doi:10.1371/journal.pone.0270616)
Supplement: S2 Appendix — (DOCX) [file pone.0270616.s002.docx]

**APPENDIX 2. Search strategies**

**MEDLINE**

| # | Search Statement | Results |
| --- | --- | --- |
| 1 | Personnel Selection/ | 12993 |
| 2 | personnel management/ or employee incentive plans/ or employee performance appraisal/ or academic performance/ | 20724 |
| 3 | career mobility/ | 11623 |
| 4 | Research Personnel/ | 16831 |
| 5 | faculty/ or faculty, medical/ or faculty, nursing/ | 33395 |
| 6 | biomedical research/ or exp genetic research/ or exp health services research/ or human experimentation/ or exp nursing research/ or exp outcome assessment, health care/ or exp pharmacy research/ or exp rehabilitation research/ or exp stem cell research/ or exp translational medical research/ | 1466055 |
| 7 | exp Behavioral Sciences/ | 252249 |
| 8 | cognitive science/ or cognitive neuroscience/ | 1267 |
| 9 | Biomedical Engineering/ | 11126 |
| 10 | Biostatistics/ | 2214 |
| 11 | Nutritional Sciences/ | 11765 |
| 12 | or/1-3 | 42278 |
| 13 | or/4-11 | 1765462 |
| 14 | 12 and 13 | 6799 |
| 15 | guideline/ | 16343 |
| 16 | benchmarking/ | 13780 |
| 17 | indicator*.mp. | 378437 |
| 18 | standard*.mp. | 1962023 |
| 19 | [productivity.mp](http://productivity.mp/). | 62650 |
| 20 | achieve*.mp. | 988610 |
| 21 | accomplish*.mp. | 101486 |
| 22 | altimetrics.mp. | 1 |
| 23 | (performance adj2 measure*).mp. | 23572 |
| 24 | or/15-22 | 3301091 |
| 25 | 14 and 23 | 2215 |
| 26 | limit 25 to (english language and humans and yr="2013 -Current") | 380 |
| 27 | limit 26 to (comment or editorial or interview or lecture or letter or news) | 42 |
| 28 | 26 not 27 | 338 |

**CINAHL**


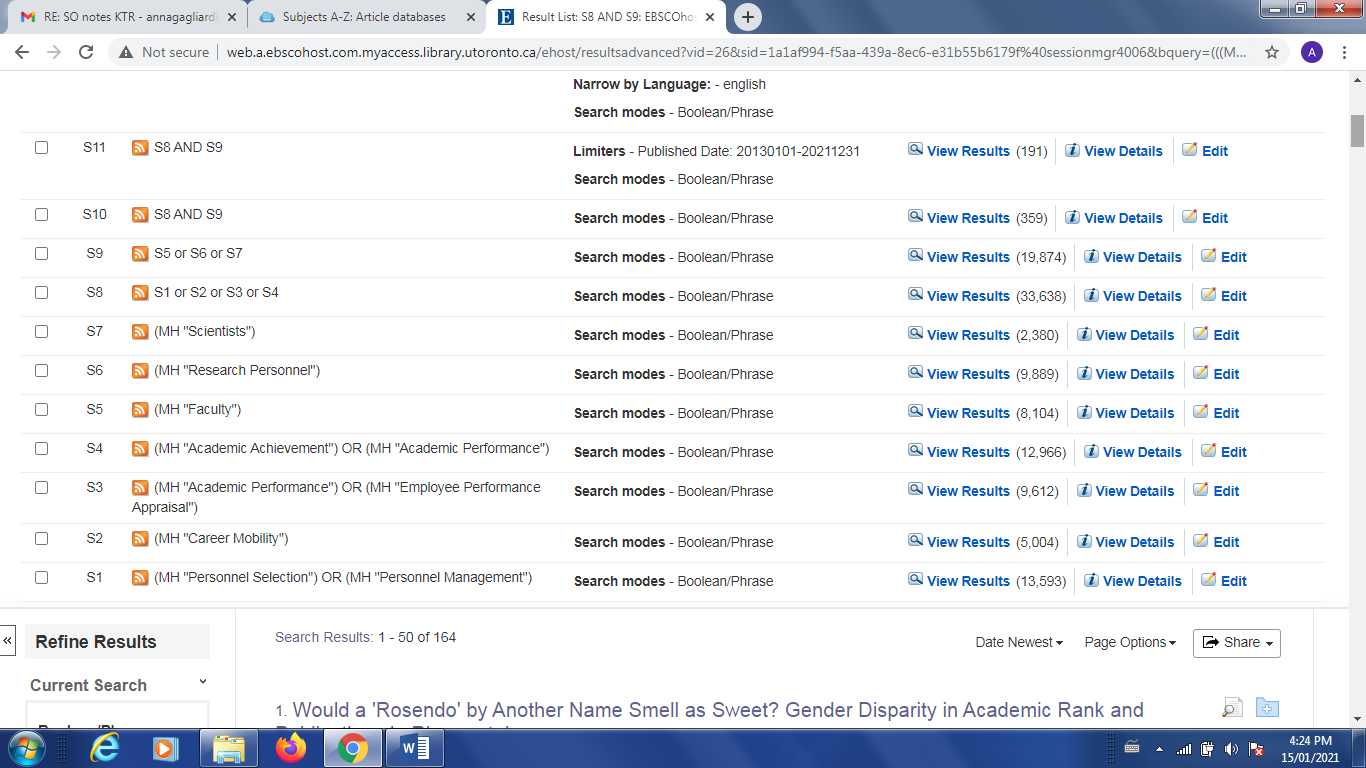


**EMBASE**

| # | Search Statement | Results |
| --- | --- | --- |
| 1 | Personnel Selection/ | 56830 |
| 2 | personnel management/ or employee incentive plans/ or employee performance appraisal/ or academic performance/ | 99961 |
| 3 | career mobility/ | 11032 |
| 4 | Research Personnel/ | 20758 |
| 5 | faculty/ or faculty, medical/ or faculty, nursing/ | 271251 |
| 6 | 1 or 2 or 3 | 108769 |
| 7 | 4 or 5 | 290180 |
| 8 | 6 and 7 | 11802 |
| 9 | limit 30 to (human and english language and yr="2013 - Current") | 1149 |

**AMED**

| # | Search Statement | Results |
| --- | --- | --- |
| 1 | Personnel Selection/ | 84 |
| 2 | personnel management/ or employee incentive plans/ or employee performance appraisal/ or academic performance/ | 372 |
| 3 | career mobility/ | 124 |
| 4 | Research Personnel/ | 0 |
| 5 | faculty/ or faculty, medical/ or faculty, nursing/ | 0 |
| 6 | biomedical research/ or exp genetic research/ or exp health services research/ or human experimentation/ or exp nursing research/ or exp outcome assessment, health care/ or exp pharmacy research/ or exp rehabilitation research/ or exp stem cell research/ or exp translational medical research/ or exp behavioural sciences | 36969 |
| 7 | 1 or 2 or 3 | 572 |
| 8 | 4 or 5 or 6 | 36969 |
| 9 | 7 and 8 | 87 |
| 10 | limit 9 to (human and english language and yr="2013 - Current") | 19 |
